# Weather radar detection of planetary boundary layer and smoke layer top of peatland fire in Central Kalimantan, Indonesia

**DOI:** 10.1038/s41598-020-79486-6

**Published:** 2021-01-11

**Authors:** Muhammad Arif Rahman, Devis Styo Nugroho, Manabu D. Yamanaka, Masahiro Kawasaki, Osamu Kozan, Masafumi Ohashi, Hiroyuki Hashiguchi, Shuichi Mori

**Affiliations:** 1 Indonesia Agency for Meteorology Climatology and Geophysics (BMKG), Jakarta, 15138 Indonesia; 2 Research Institute for Humanity and Nature, Kyoto, 603-8047 Japan; 3 Center for South East Asian Studies, Kyoto University, Kyoto, 606-8501 Japan; 4 Department of Information Science and Biomedical Engineering, Kagoshima University, Kagoshima, 890-8580 Japan; 5 Research Institute for Sustainable Humanosphere, Kyoto University, Uji, 611-0011 Japan; 6 Japan Agency for Marine‐Earth Science and Technology, Yokosuka, 237-0061 Japan

**Keywords:** Environmental sciences, Natural hazards

## Abstract

During the dry period of August–October 2015, a C-band Doppler weather radar of the BMKG station in a fire-prone peatland area, Palangka Raya, detected echoes with reflectivity values between − 19 and + 34 dBZ at a height below 2–3 km and a slant range of 100 km. The MERRA-2/NASA atmospheric reanalysis database is used to obtain the vertical profiles of refractive index and equivalent potential temperature of the air. The temporal variation of the radar image is due to the tropical diurnal cycle of planetary boundary layer formation, which is consistent with the results of the database analysis. The echo images are discussed in terms of Bragg scattering of microwaves at the top of the planetary boundary layer. Weather radar monitoring of the fire smoke layer-top images has a potential feasibility to support real-time management of peatland fires.

## Introduction

**Figure 1:**
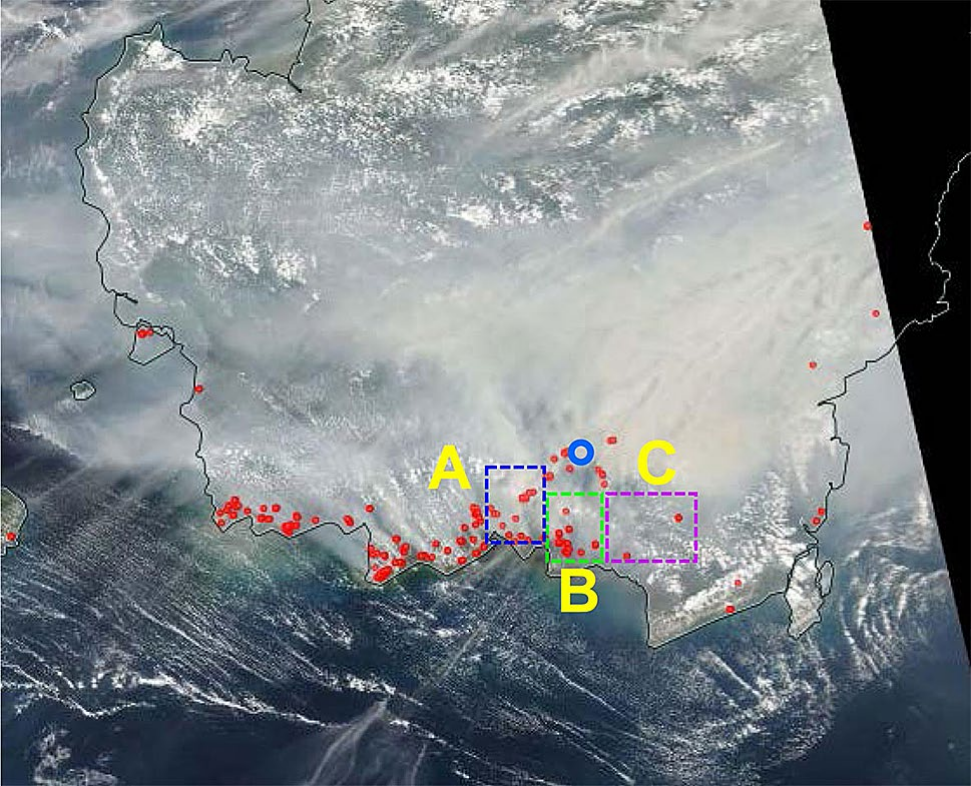
Terr and Aqua/MODIS/NASA true-color image of 15 October 2015 in Kalimantan, Indonesia. The imagery resolution is 250 m. The blue circle indicates the location of Palangka Raya. Red dots are MODIS fire hotspots. Study districts A, B and C are marked by the three squared areas. (Power Point 2019).

Ground-based weather radar reveals the tropical diurnal cycle in land-sea migration of the active precipitating convective clouds over the Indonesian maritime continent^1^. Here we propose weather radar detection of the mixed planetary boundary layer (PBL) top, formation of which is due to either the diurnal cycle or capping smog produced from peatland fires during the non-rainy season in Central Kalimantan, Indonesia. The number of fire hotspots reported by MODIS during August–October 2015 is 28,754 in Kalimantan. Large-scale forest/peatland fires burning over most notably Indonesia released 11.3 Tg CO_2_ per day during September–October 2015, emissions from these fires exceed the fossil fuel CO_2_ release rate, 8.9 Tg CO_2_ per day of the European Union, EU28^2^. Figure 1 is a true-color image from MODIS/Terra and Aqua/NASA satellites above Kalimantan on 15 October 2015 during the El Niño event. The image shows a thick yellowish puff of smoke that originates from forest/peatland fires. The atmospheric stratification is responsible for capping air contaminated with aerosol and water vapour produced from peatland fires by suppression of the atmospheric convection.

The first observation of fire smoke by a lidar and a 3.2-cm wavelength radar was conducted in the middle latitude by Banta et al.^3^ McCarthy et al.^4^ reviewed the capacity for aiding fire management by using radar echoes as a proxy for wild and prescribed fire pollution. Price et al.^5^ examined the maximum height, width, and areal trail of large-particle plumes from fires in Australia. Tsai et al.^6^ used a W-band radar and lidar to investigate forest fires. Its application is informing firefighter safety by detecting and warning of convective outflows because high winds generate fire storms that move rapidly through the crowns of trees. The radar uses in this capacity are apparent in Arizona^7^ and Colorado^8^, USA. Near ground in such mid-latitude regions, the PBL affects the aerodynamic behaviours of fire-produced particulate matters, nitrogen oxides and humidity.

The radar echoes arise from scattering or reflection by eddies with sizes of half a radar wavelength as indicated by the refractive index gradient. The combined processes of evaporation and transpiration change the radio index of refraction, which is given by a function of atmospheric pressure, the partial pressure of water vapour, and temperature. The diurnal cycle is the most dominant PBL phenomenon near the coastlines in the tropics, particularly in Indonesian islands^1^. Its larger horizontal scale and thicker vertical stratification covered by a stable layer are different from those in mid-latitudes^9^. The tropical diurnal cycle induces PBL formation near the ground in the morning after sunrise, which ascends to a few km high. It causes radar echoing due to the Bragg scattering processes since its top is associated with eddies in the gradients of temperature and humidity. Hashiguchi et al.^10^ reported those features using an L-band radar installed near Jakarta, Indonesia. There have been other studies on radar observation of the PBL^11,12^.

In this article, we report characteristic echoes of the C-band Doppler radar measurements during the El Niño event 2015. In El Niño the western Pacific including Indonesia is extremely dry, and the diurnal cycle of PBL is associated with less active clouds during the daytime. These weather conditions cause serious peatland fires. The weather radar of the Tjilik Riwut BMKG station (WMO station name: WAGG, 2.224°S, 113.946°E, 10 m a.s.l.) has been operating since 2010 in a fire-prone peatland area, Palangka Raya (PKY), Central Kalimantan, Indonesia. This location is suitable for monitoring both the tropical diurnal cycle of PBL formation and the dynamic behaviour of forest/peatland fires. The echo image intensity due to PBL became appreciable during daytime. Fire smoke layer tops were also seen in real-time radar images.

## Results

Figure 1 shows the study districts on the true-color MODIS image of 15 October 2015 in Central Kalimantan, Indonesia. The blue circle is the location of the weather C-band radar at PKY, covered by yellow puff smoke. The MODIS fire hotspots shown with red dots are seen in the southern area from which seasonal wind blows towards the northern area.

### Radar echo intensity around Palangka Raya

**Figure 2:**
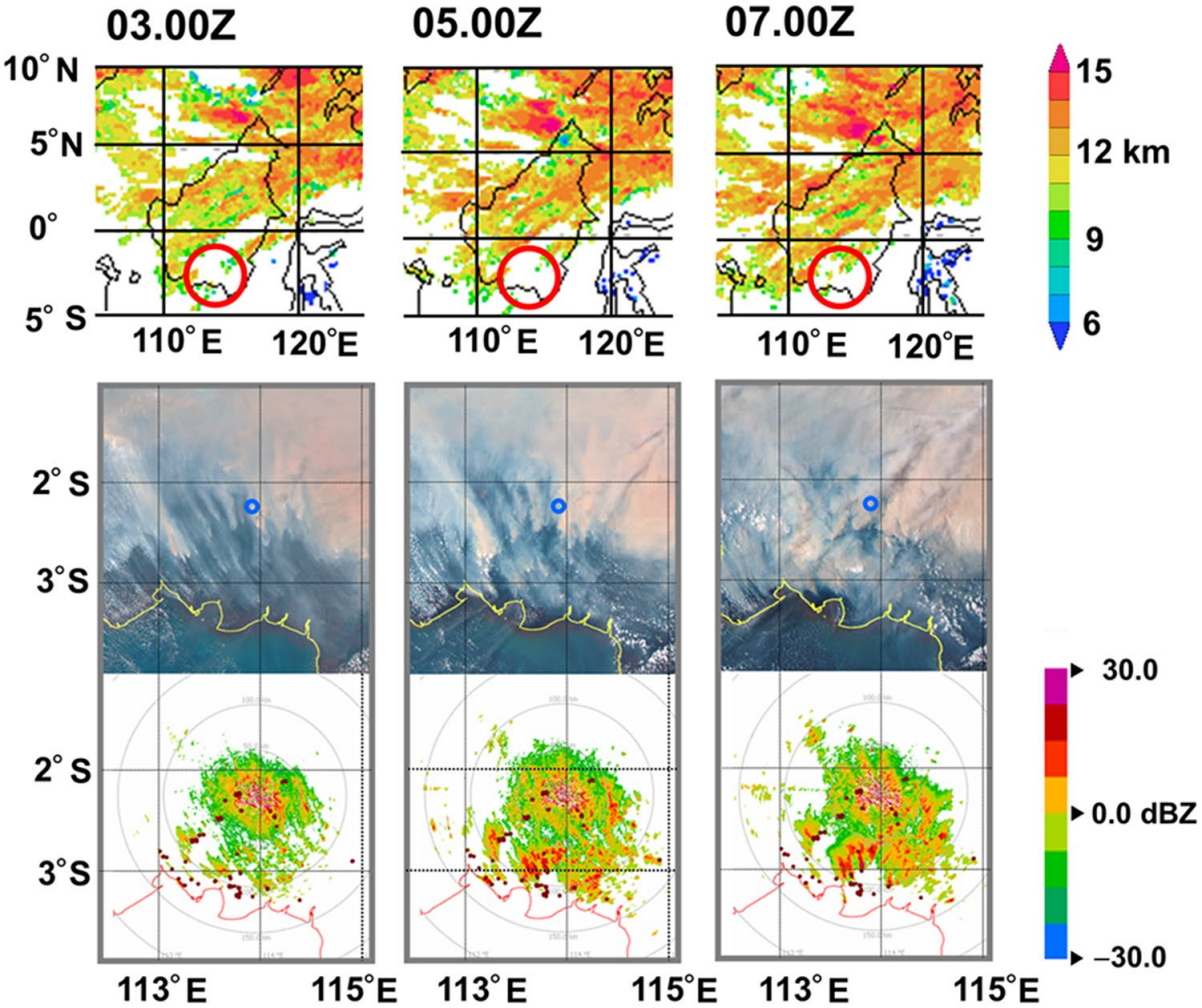
Lower: Radar echo images observed on 15 October 2015 from 03Z (10LT) to 07Z(14LT) with PPI = 0.5˚. Radar is located at the Tjilik Riwut BMKG station of Palangka Raya. Red dots are MODIS fire hotspots. Note that radar images show the evolution of the planetary boundary layer and fire plume trails. Middle: Corresponding AHI/HIMAWARI-8/JMA satellite images. The blue circle indicates the location of Palangka Raya. Upper: Cloud-top height and optical thickness estimates by RISH/KU. The white non-cloudy regions in the red circles cover the study areas. (Rainbow 5, https://www.leonardocompany.com/en/products/rainbow-5-application-software), (Power Point 2019).

In this work, for simplicity, we present one day radar data. Similar echo scattering images were obtained for the entire dry periods in 2014 and 2015. Hereafter, all data in this article are for 15 October 2015, unless otherwise stated. Figure 2 shows the plan position indicator (PPI) images at 03Z–07Z (10LT–14LT) within a slant range of 100 km at an elevation angle of 0.5°, which covers the peatland areas across the districts of Katingan, Pulang Pisau, Palangka Raya, East Kotawaringin and Kapuas. Corresponding AHI/HIMAWARI-8/JMA satellite true-color images in Fig. 2 show wide-spread smoke and trails of fire plume. Detailed evolution of the echo scattering images is shown in Supplementary Fig. S1 on line, in which the echo signal became strong at noon, 05Z, and weak in the late afternoon, 10Z. We also found that the radar echoing layer stayed below 2–3 km height during the daytime. The radar echo comes from the top of the PBL as will be discussed below. The echoing does not come from cloud precipitation because the cloud-top heights estimated by Research Institute for Sustainable Humanosphere (RISH) of Kyoto University (KU) in Fig. 2 do not indicate the existence of a convective cloud in the study districts in Fig. 1. To ensure that there are no echo disturbances from rain during the daytime radar measurements, the brightness temperature of cloud top is shown in Supplementary Fig. S2 on line, in which the black body temperature observed by AHI/HIMAWARI-8 was 266 − 294 K, indicating no active clouds.

**Figure 3:**
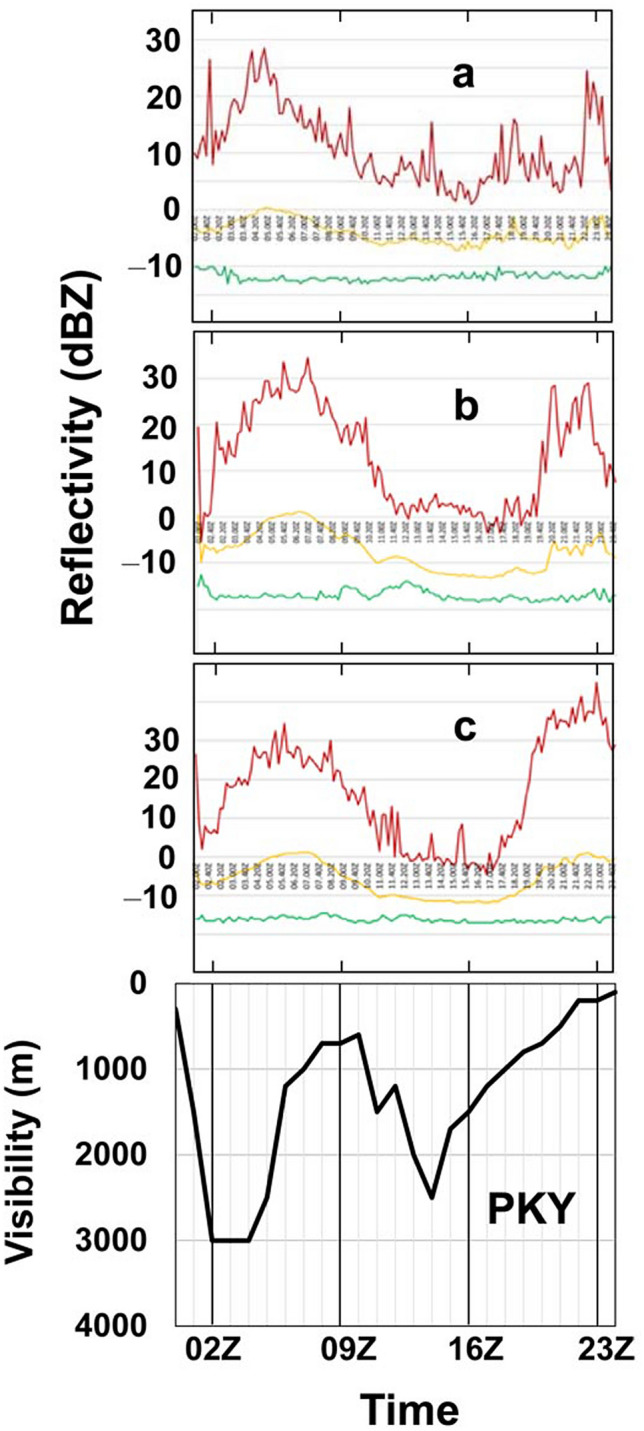
**(a, b, c**) Temporal variations of the maximum (red), mean (yellow) and minimum (green) signal intensities of radar echoes in the districts A, B and C of Fig. 1, respectively. **PKY**: Reversely ordered visibility data at Palangka Raya on 15 October 2015. (EXCEL 2019, Power Point 2019).

For the study districts, A, B and C in Fig. 1, the timeseries of the echo levels in Fig. 3 show the maximum (red), mean (yellow) and minimum (green) signal intensities. Here the interference noise from frequencies outside the radar C-band after 18Z is omitted. The minimum signal intensities are in the range of − 19 – − 10 dB*Z*, which are close to the noise floor. In all districts, the echo signal starts increasing at 02Z and peaks at 05Z with a maximum value of 28–34 dBZ and a mean value of 0–1 dBZ during the daytime. The wind direction speculated from plume trails in the HIMAWARI-8 satellite images of Fig. 2 is southeasterly. Trail features of radar signal appear mainly in the southern areas, the origins of which are fire hot spots. Since PKY is located downwind of the hotspot districts, the air in the PKY area became contaminated with smoke in the afternoon and at night.

In Fig. 3 the reflectivity of the districts, B and C, recovered for 20Z–23Z. During that time, since temperature is low with high humidity and slow wind speed, smoke aerosol particles grow to larger size by condensation of water onto them. This growth of aerosol size was previously discussed using a correlation between airport visibility and weather data in PKY by Iriana et al.^13^.

### Calculation of radar refractive index from objective analysis data

Radar echoes arise from scattering or reflection at a layer with eddies, the size of which is half of a radar wavelength, in the atmospheric dielectric constant or index of refraction, $n$ ^14,15^. The radio index of refraction for the lower neutral atmosphere is given approximately by,

1 $$n-1=\frac{3.73\times {10}^{-1}e}{{T}^{2}}+\frac{77.6\times {10}^{-6}p}{T},$$

where *e* is the partial pressure of water vapour in units of hPa, $p$ the atmospheric pressure in hPa, and $T$ the absolute atmospheric temperature in K^16^. The first term is more important in the lower troposphere because of high humidity. The second term, the dry air contribution, is important from the mid-troposphere up to the stratopause.

**Figure 4:**
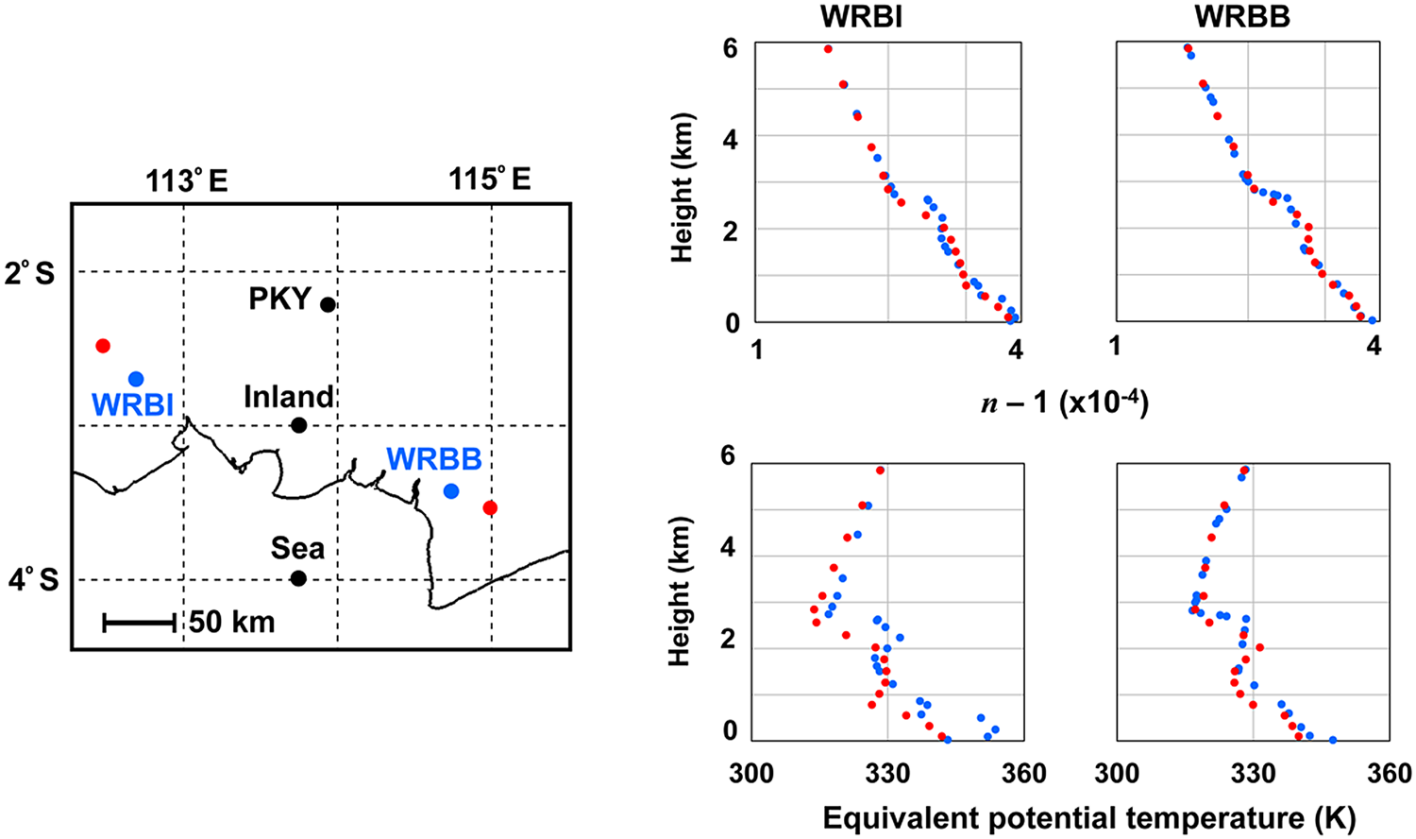
(Left) Blue dot: locations of BMKG stations, WRBI and WRBB. Red dot: MERRA-2/NASA database locations. Two black dots, “inland” and “sea”, are for inland and on-sea locations. PKY stnads for the Tjilik Riwut BMKG station in Palangka Raya, (Right) vertical profiles of the refractive index, *n*, and equivalent potential temperature. Blue dot: radiosonde data. Red circle: derived from MERRA-2/NASA database. At 00Z on 13 September 2018. The scale for refractive index is logarithmic. (Visual Studio 2017, EXCEL 2019, Power Point 2019).

We calculated $n$ in Eq. (1) with the MERRA-2/NASA database for *p*, *e,* and *T*. To confirm the validity of our procedure, we chose two BMKG stations in Central Kalimantan, WRBI (Pangkalan Bun: location 2.70°S, 112.70°E) and WRBB (Banjar Baru: 3.43°S, 114.75°E) where the radiosonde data of the Wyoming University database are available. The corresponding MERRA-2 grid points are (2.50°S, 112.50°E) and (3.50°S, 115.00°E), respectively. These locations are shown in Fig. 4. The equivalent potential temperatures and refractive indices from the two databases at 00Z of 13 September 2018 are compared in Fig. 4. The vertical profiles derived from the MERRA-2 database reveal singular levels corresponding to the PBL tops returning radar echoes. We found that same-day values derived from the MERRA-2 and radiosonde databases are available for 112 days in February − September 2018. These two values are in good agreement with each other as seen in Fig. 4. The accuracy of the dataset was reported by Luo et al.^17^ In the following section, the MERRA-2 database is used to analyze PBL formation in the study districts where the radiosonde data are not available.

### Temporal variation in the vertical profile of the index of refraction

**Figure 5:**
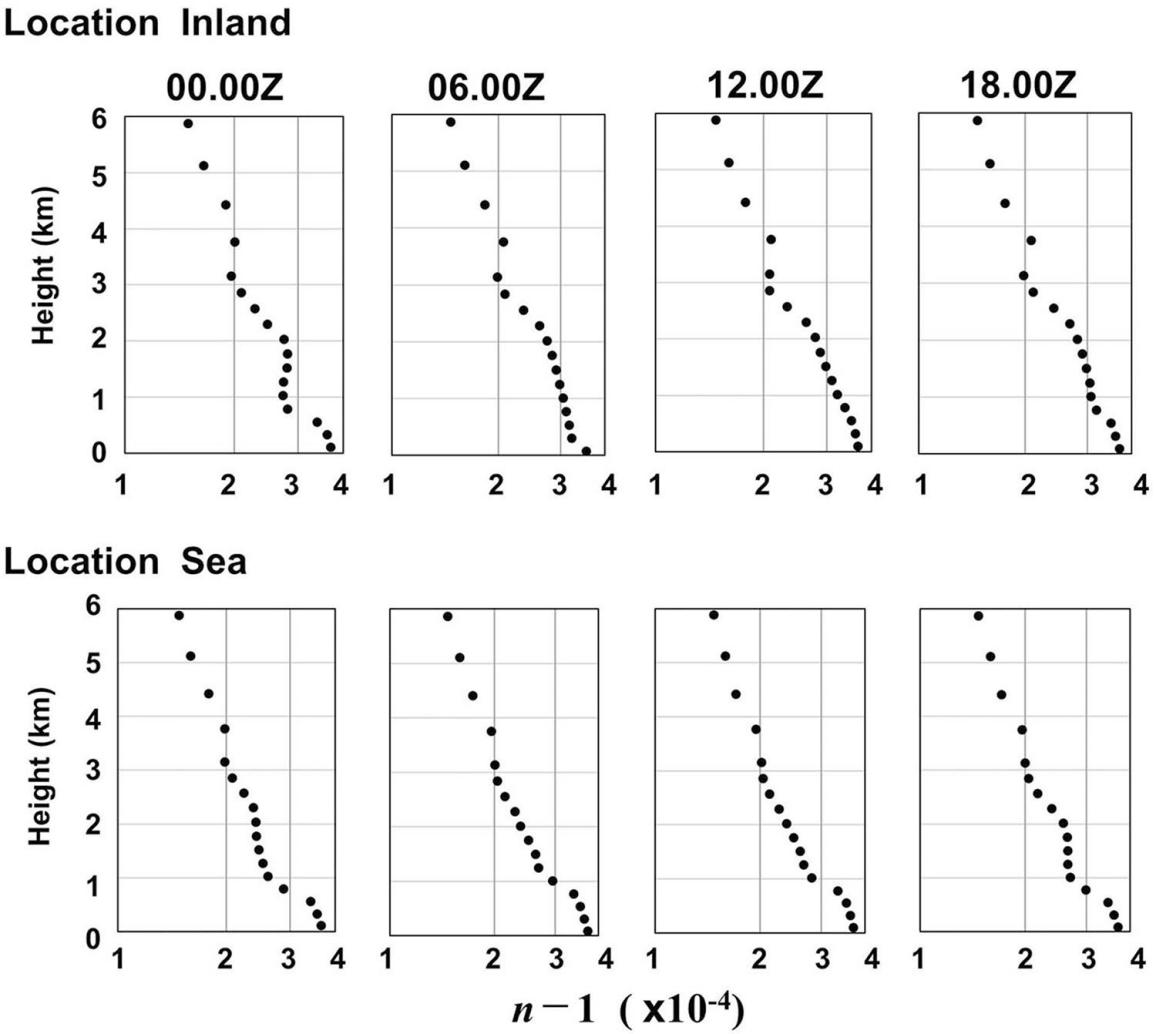
Diurnal changes of refractive indices calculated as a function of height, time and location using MERRA-2/NASA database of 15 October 2015. Locations are “inland” and “sea” of Fig. 4. (EXCEL 2019, Power Point 2019).

To investigate the contribution of PBL formation to radar echoes on 15 October 2015, we calculate the vertical profiles of ($n-1$) at two locations in Fig. 4: an inland point of the southern Central Kalimantan and a sea point using the MERRA-2 objective analysis data that have representability and accuracy to reveal an altitude range involving mixed layer top and refraction/scattering level. On the inland point at 00Z in the morning, steep profile changes in $(n-1)$ are seen at both $h$ = 2 − 3 km and $h$ = 0.6 km in Fig. 5. After 06Z in the afternoon, the lower layer goes up to form the stable PBL layer until 12Z at sunset. Then, after 18Z in the midnight, a change in $(n-1)$ is seen at the lower height due to the cooling effect of the ground. See Supplementary Figs. S3, S4 and S5 on line for detailed profiles. This tropical diurnal cycle in the formation of the PBL top with refractive eddies induces the Bragg scattering of radio wave during the daytime^18,19^.

These results explain the temporal variations of the radar echo images in Fig. 2, which were distributed mainly within 50 km, and the intensity changes in Fig. 3 by the evolution of PBL construction in the inland area. The temporal variation of the maximum intensity is typical of the PBL diurnal cycle in the dry period of the maritime continent^20,21^. For tropical peatland fires occurring before around noon, the lower atmosphere is capped by a dry and stable layer, which works as a duct for smoke and causes the Bragg scatter for the weather radar. During prescribed fires in the daytime, any insects making an "angel echo" were not observed visually, but should be examined in subsequent studies on radar application to aeroecology^22^.

In contrast, the vertical profile change of $(n-1$) above the sea point is small for the entire day, as seen in Fig. 5 and Supplementary Fig. S5 on line. The difference in the temporal change between the inland and on-sea locations is characteristic of the Indonesian maritime continent^1^. This diurnal behaviour of the PBL top appears every day in Central Kalimantan during the dry fire period.

### Temporal variations in ground aerosol concentration and visibility at Palangka Raya

The increase of aerosol concentration causes turbidity of the atmosphere with reduced visibility that is in inverse proportion to aerosol optical depth (AOD)^23^. Ground aerosol observation using an AERONET sun photometer was performed at the Tjilik Riwut BMKG station in PKY. During the dry period, August**–**October 2015, AOD at 500 and 870 nm increased up to 6 due to fire aerosol as seen in Supplementary Fig. S6 on line. For the temporal variation during 00Z–08Z on 15 October 2015 in the same figure, AOD at 870 nm started increasing at 05Z and reached four at 08Z. Because of the thick aerosol density, the sun photometer stopped observation after 08Z due to diminished solar light.

At the Tjilik Riwut BMKG station, we measured the hourly variation of visibility, which varied from 300 to 3000 m as shown in Fig. 3. After hazy air masses reached the PKY area at 05Z from the southern peatland area, visibility decreased to 700 m at 09Z. As seen in Supplementary Fig. S7 on line, a strong correlation (*R*^2^ = 0.93) between one-hour averaged values of airport visibility and sun-photometer AOD at 870 nm for 00Z–08Z indicates that we observed in PKY the hazy air mass influx from the fire hotspot areas.

In the nighttime after surface fires ceased, the visibility increased up to 2000 m due to flow-in of the airmass with low aerosol concentration, and then again decreased to 300 m at 23Z just before sunrise. Since humidity increases with lowering temperature under slow wind speed conditions in the nighttime, smoke aerosol particles grow to a larger size and hence visibility is reduced due to light scattering. This process is enhanced through the PBL capping of the ground air. Just after sunrise at 24Z, visibility suddenly recovered. This temporal variation is characteristic of the tropical weather^13^. Supplementary Fig. S8 on line shows the temperature and humidity data at Palangka Raya Airport, in which humidity was > 90% and temperature < 25 °C at midnight and early morning until the sun rose.

## Discussion

About the equatorial PBL formation, Hashiguchi et al.^10^ reported two types of L-band radar echo structures that appeared systematically with diurnal variations on clear days. The first type is the appearance of a strong echo layer ascending from 0.3 km in the morning to 3–5 km in the afternoon. This is identified as a diurnal cycle of the mixed PBL top. The second type is an echo layer appearing at 2–3 km heights during nighttime, which is coincident with humidity gaps. Especially in fire fields, through the direct aerosol effect or radiative forcing from aerosol-radiation interactions, the ground temperature becomes colder during the day and warmer at night because of shortwave and longwave radiation properties of aerosol pollutants, respectively. Thus, severe aerosol pollution caused by peatland fires leads to the intensification of stable stratification near the surface throughout the day. Aerosols heat the ground at night, whereas aerosols cool the air at the same time. In addition, based on the measurements of visibility in Fig. 3 and AOD in Supplementary Fig. S6 on line, the heavily contaminated air mass flowed into the PKY region after 05Z with concentration peak at 08Z–10Z. However, Fig. 3 shows the maximum signal intensity of radar echo started increasing at 02Z and peaked at 05Z, suggesting a time lag between the aerosol concentration in PKY and the radar echo intensity in the study districts of Fig. 1. Thus, the widespread radar echo images of this work are caused by the diurnal cycle of PBL formation in the fire field during the daytime, which is enhanced by hazy air mass. From night to early morning 18Z − 23Z, the radar echo intensities as well as the visibility in reverse order or the concentration of scatterer monotonically increased, as seen in Fig. 3. Since relative humidity increases with low temperature under slow wind speed conditions, the most likely possibility of the observed nighttime reflectivity is due to the humidity gaps caused by condensation of water onto smoke particulates.

**Figure 6:**
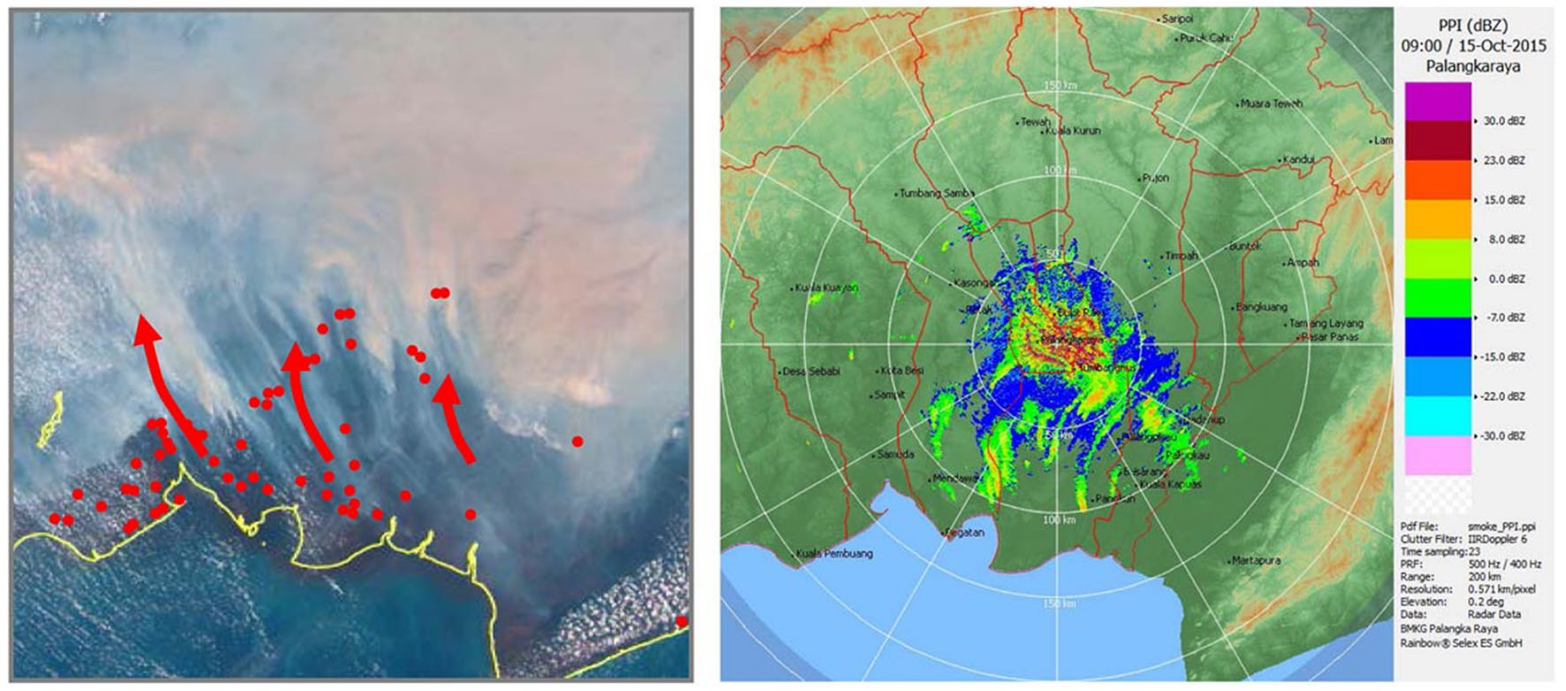
Right: Radar echo image at 09Z(16LT), 15 October 2015. Left: Smoke plumes are observed by AHI/HIMAWARI-8/JMA satellite. Red dots are MODIS/NASA fire hotspots. Red arrows are forward trajectories at 1000 m altitude using HYSPLIT MODEL/NOAA. (Rainbow 5, https://www.leonardocompany.com/en/products/rainbow-5-application-software), (Power Point 2019).

The fire smoke layer-top trails in the radar echo images of Figs. 2 and 6 are seen in the daytime, which extend from the MODIS hotspot points towards the northern areas. Corresponding AHI/IMAWARI-8 true-color images in the same figures also show trails of fire smoke layer-tops. The echo intensities varied from − 15 to + 22.5 dBZ during the PPI scanning and the maximum intensity peaked at 05Z − 07Z in Fig. 3. To confirm the origin of these smoke layer-top trails, we analyzed smoke trajectories starting from the hotspots, as shown by the red arrows in Fig. 6, in which three trajectories are projected in the study areas with use of the forward trajectories for 02Z − 08Z at 1000 m altitude using HYSPLIT MODEL/NOAA. Comparing these trajectories with the smoke layer-top trails of the radar echo images, they match each other. According to Zender et al.^24^, the smoke flows with the prevailing southeast surface winds at a speed of 3 − 4 m s^−1^ in the dry season of Central Kalimantan, and forms ovoid plumes whose mean length, height, and cross-plume width are 41 km, 0.7 km, and 27% of the plume length, respectively. These reported sizes agree with the trail features observed in Figs. 2 and 6. Price et al.^5^ reported that 45% of the plumes were radar-detected for large fires (> 100 k ha) closer than 50 km from the radar in south-east Australia. Plume heights they measured are median 3 km for the wild fires and 1.7 km for the prescribed fires. Melnikov et al. ^25^ reported plume reflectivity around 5–10 dBZ as observed by a dual polarisation S-band WSR-88D radar during a wild fire. In spruce forest fire that occurred in south-central Alaska, relatively high reflectivity (20–25 dB*Z*) was observed by a WSR-88D radar in the smoke plumes near the head of rapidly moving fires^26^. Transport and dispersion of smoke plume from the prescribed burns were reported. According to Kusumaningtyas et al.^27^, the aerosol size distribution during the burning seasons at PKY is bimodal with peaks at 0.2 and 4.0 μm. The lower-sized aerosol is produced from peatland smoldering smokes.

McCarthy et al.^4^ reviewed the sources of the radar scatterer for the plume of mid-latitude fires, which are ash or pyrometeors. Considering the sensitivity of the C-band radar observations the Rayleigh approximations predict that radar detects only large particles > 100 μm^5^. Jones et al.^28^ eliminated aerosol-size smoke (less than 10 μm) as a candidate scatterer in their dual-polarisation radar observations of a flat fire. Using a W-band radar Tsai et al*.*^6^ reported reflectivity of − 30 dBZ when little or no lofted debris was observed, and the smoke plume above the radar site was only faintly visible. They hypothesised that for these moist fires, the smoke particles serve as condensation nuclei permitting observation by the radar. A study of fires in Alaska showed that some of the enhanced brightness in synthetic-aperture radar imagery was due to increased ground moisture rising to the surface after the fire^29^. The X-band radar images of high-intensity canopy fires in Victoria, Australia came from pyrometeors in the moist convective plumes^30^. The larger pyrometeors fallout of the plume once the turbulence and vertical velocities of the plume decrease.

Based on the discussion above, the present C-band radar of the Tjilik Riwut BMKG station cannot detect echoes due to the Rayleigh scattering of smoke particles. The large values of radar reflectivity shown in Fig. 3 could correspond to raindrops, insects, or the layered turbulence caps. No rain was observed during the daytime radar measurements. If insects in the turbulent air behave as tracers^22^, their echoes could be important for watching the PBL top and fire plume behaviours. Although insects might be tracers for atmospheric convection, their presence could not be confirmed simultaneously when the BMKG radar echoes appeared during the daytime fires. In this study, we hypothesise that the weather radar detects the Bragg scattering signals from the mixed layer top capping fire smokes since the windward edge boundaries are associated with eddies in temperature and humidity. Although Bragg scatter is still a hypothesis without simultaneous turbulence measurements, other observations suggest broad wavenumber spectra of turbulence including the Bragg wavelength (2.66 cm for 5640 GHz radar)^10,15^. Weather radars (S and X band) detected smoke plumes from the industrial fire in Montreal, Canada by Rogers et al.^31^ who hypothesised the Bragg scattering caused by refractive index irregularities.

## Summary and conclusion

In a fire-prone equatorial peatland district, Palangka Raya of Indonesia, we have performed radar echo measurements during the dry season using a C-band precipitation radar*.* With a combination of measurement and analysis of data for airport visibility, atmospheric optical density, atmospheric reanalysis database, satellite image and forward trajectory calculation, the observed radar echo images are interpreted as a lower atmospheric structure, the tropical planetary boundary layer characteristic of the fire-prone tropics. Its daytime construction is associated with the diurnal variation, whereas night-time one is enhanced through the effect of fire aerosols. We also show the plan position indicator echo of the weather radar can be operationally useful for real-time management of large-scale forest and peatland fires.

## Methods and data sources

### Weather radar specification

(https://www.bmkg.go.id/cuaca/citra-radar.bmkg) The C-band Doppler radar was installed in a tall tower of 15 m high. The radar specification is as follows: manufacturer SELEX, model Meteor 600C, frequency 5.640 GHz, single horizontal polarisation, beam width 1°, pulse repetition frequency (PRF) 250–1200 Hz and a magnetron type transmitter. The scanning strategy was VCP21 with low elevation angles: 0.2, 0.5, 1.0, 1.5, 2.4, 3.4, 4.3, 6.2, 10°, 14°, and 19.5°. This scanning strategy used dual PRF (450 and 600 Hz) with a pulse width of 0.8 µs for sensitive scan strategy. The PPI products with a single elevation at 0.5° that detected radar echoes close to the surface were analyzed to retrieve reflectivity data. Note that this usage of a weather radar for non-rainy air echoes is not standard. After adjusting the pulse compression to improve the horizontal resolution of raindrop echoes with suppressing noise for standard usage, the mixed-layer top echoes disappeared. An analysis tool, Rainbow 5 weather application, was used to monitor images. (https://www.leonardocompany.com/en/products/rainbow-5-application-software).

### Air pollution data from satellite, radiosonde and ground base

To calculate the radio refractive indices at PKY and other locations in Central Kalimantan, MERRA-2 reanalysis database was used. (M2I3NPASM_5.12.4, https://disc.gsfc.nasa.gov/ datasets/) True sRGB images of the AHI/HIMAWARI-8/JMA satellite were obtained from National Institute of Information and Communications Technology (http://himawari8.nict.go.jp/ja/himawari8-image.htm). The products of cloud top height and optical thickness from RISH of Kyoto University (version 3, http://database.rish.kyoto-u.ac.jp/arch/ctop/index_e.html). Fire hotspot data were provided by Terra and Aqua/MODIS/NASA satellite. The imagery was visualised in Worldview and the Global Imagery Browse Services. (https://worldview.earthdata.nasa.gov/) The radiosonde data were from Wyoming University database (http://weather.uwyo.edu/upperair/sounding.html). Air quality and visibility data of PKY were provided by AERONET/NASA (Level 2, http://aeronet.gsfc.nasa.gov) and the BMKG station in Palangka Raya of Indonesia (http://dataonline.bmkg.go.id/home), respectively. Forward trajectory calculations were performed using HYSPLIT MODEL/NOAA (https://ready.arl.noaa.gov/hypub-bin/trajtype.pl).

## Supplementary information

Supplementary Information 1.

## Data Availability

Data during the study are available in https://drive.google.com/drive/u/0/folders/1I6THf6jEXNIRtCXy2olxR4fHz-jQLko0.
