# Supplementary material for: Weather radar detection of planetary boundary layer and smoke layer top of peatland fire in Central Kalimantan, Indonesia
Source: Sci Rep. 2021 Jan 11;11:367. doi: 10.1038/s41598-020-79486-6 (PMC7801614; doi:10.1038/s41598-020-79486-6)
Supplement: Supplementary file 1 — Supplementary Information 1. [file 41598_2020_79486_MOESM1_ESM.pdf]

Supplementary Information

Weather radar detection of planetary boundary layer and smoke layer top of peatland fire in Central Kalimantan, Indonesia

Muhammad Arif Rahman[1*], Devis Styo Nugroho[1], Manabu D. Yamanaka[2], Masahiro Kawasaki[2]✉, Osamu Kozan[2,3], Masafumi Ohashi[4], Hiroyuki Hashiguchi[5] and Shuichi Mori[6]

[1]Indonesia Agency for Meteorology Climatology and Geophysics (BMKG), Jakarta 15138, Indonesia. [2]Research Institute for Humanity and Nature, Kyoto 603-8047, Japan. [3]Center for South East Asian Studies, Kyoto University, Kyoto 606-8501, Japan. [4]Department of Information Science and Biomedical Engineering, Kagoshima University, Kagoshima 890-8580, Japan. [5]Research Institute for Sustainable Humanosphere, Kyoto University, Uji 611-0011, Japan. [6]Japan Agency for Marine-Earth Science and Technology, Yokosuka 237-0061, Japan. ✉ email: kawasaki@moleng.kyoto-u.ac.jp

1. Diurnal cycle of the radar echo images. Radar echo images in Fig. S1 show the diurnal cycle of the planetary boundary layer and fire smoke layer top. The radar was located at the Tjilik Riwut BMKG Station of Palangka Raya. The time lag between UTC and the local time is seven hrs.

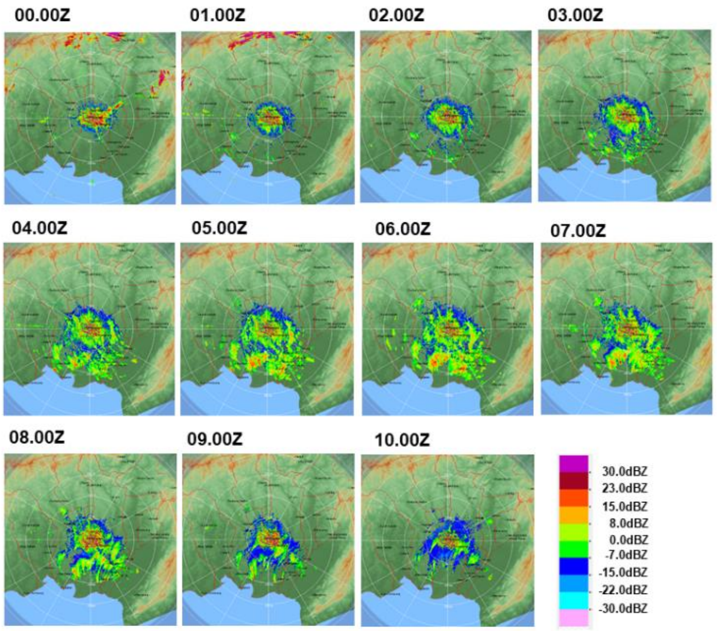

Figure S1. Radar echo images observed on 15 October 2015 from 00.00Z to 10.00Z with PPI = 0.5˚. (Rainbow 5, https://www.leonardocompany.com/en/products/rainbow-5-application-software), (Power Point 2019)

2. Cloud top height and optical thickness in the study regions

In Fig. S2, the brightness temperatures of Band 14 at 11 μm are 266- 294 K in the regions marked with the red circles on 15 October 2015, which are the study regions of Main Text Fig. 1. These temperatures

correspond to the fact that the cloud-top height analysis in Main Text Fig. 2 shows no image of a convective cloud.

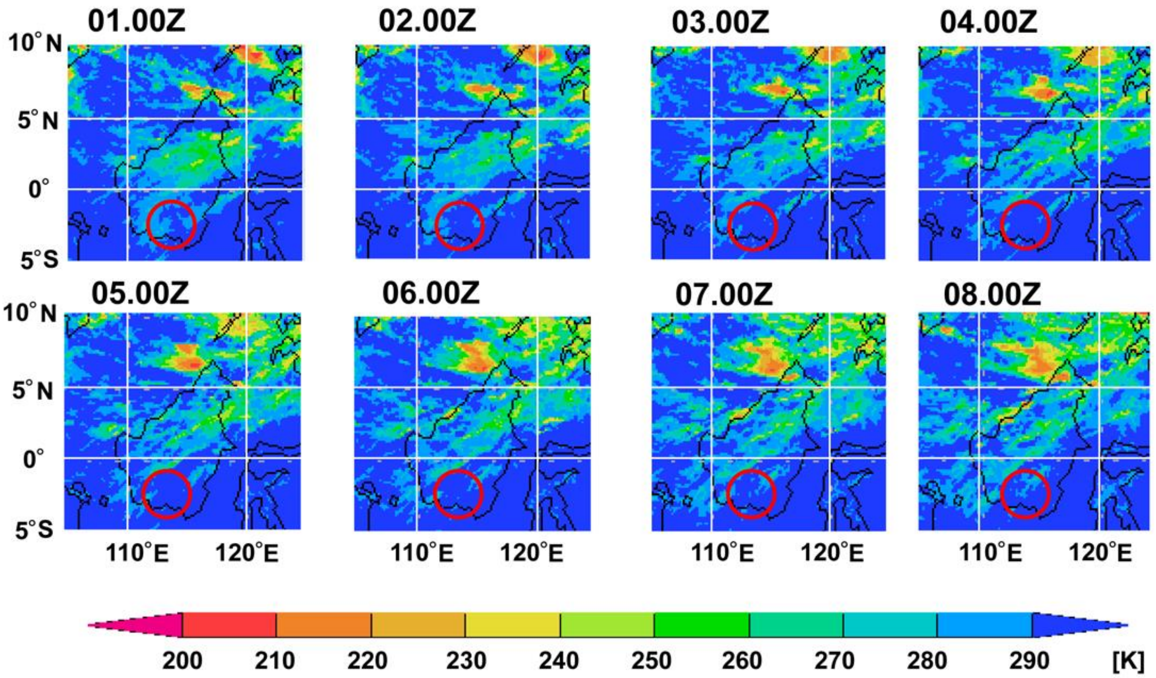

Figure S2. Brightness temperature images on 15 October 2015, which are supplied by Research Institute for Sustainable Humanosphere (RISH) of Kyoto University from HIMAWARI-8/JMA satellite data of Band 14. RISH of Kyoto University (version 3) http://database.rish.kyoto-u.ac.jp/arch/ctop/index_e.html). (Power Point 2019)

3. Vertical profiles of refractive index, temperature and equivalent potential temperature

The refractive indices and equivalent potential temperature are calculated from the MERRA-2/NASA database for the inland and sea locations in Fig. S3. The refractive indices are shown in logarithmic scale in Figs. S4 and S5 for clarity, respectively. The figures for the inland location show the ascending and descending behaviour of the planetary boundary layer top, while not for the sea location.

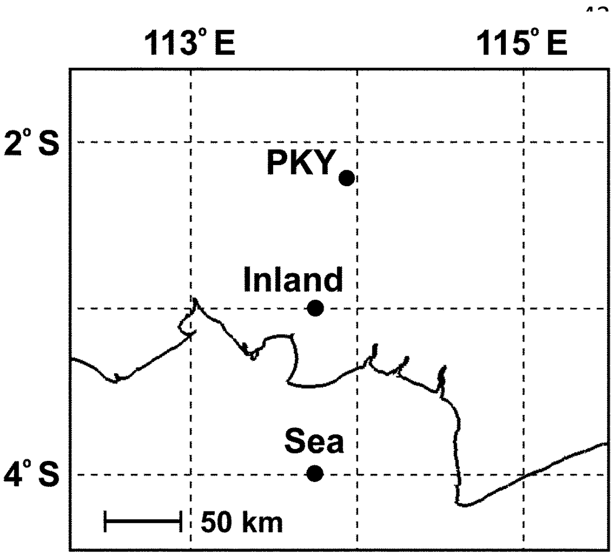

Figure S3. Locations of inland and sea points for the MERRA2/NASA reanalysis data. PKY stands for the Tjilik Riwut BMKG Station in Palangka Raya (Visual Studio 2017, Power Point 2019)

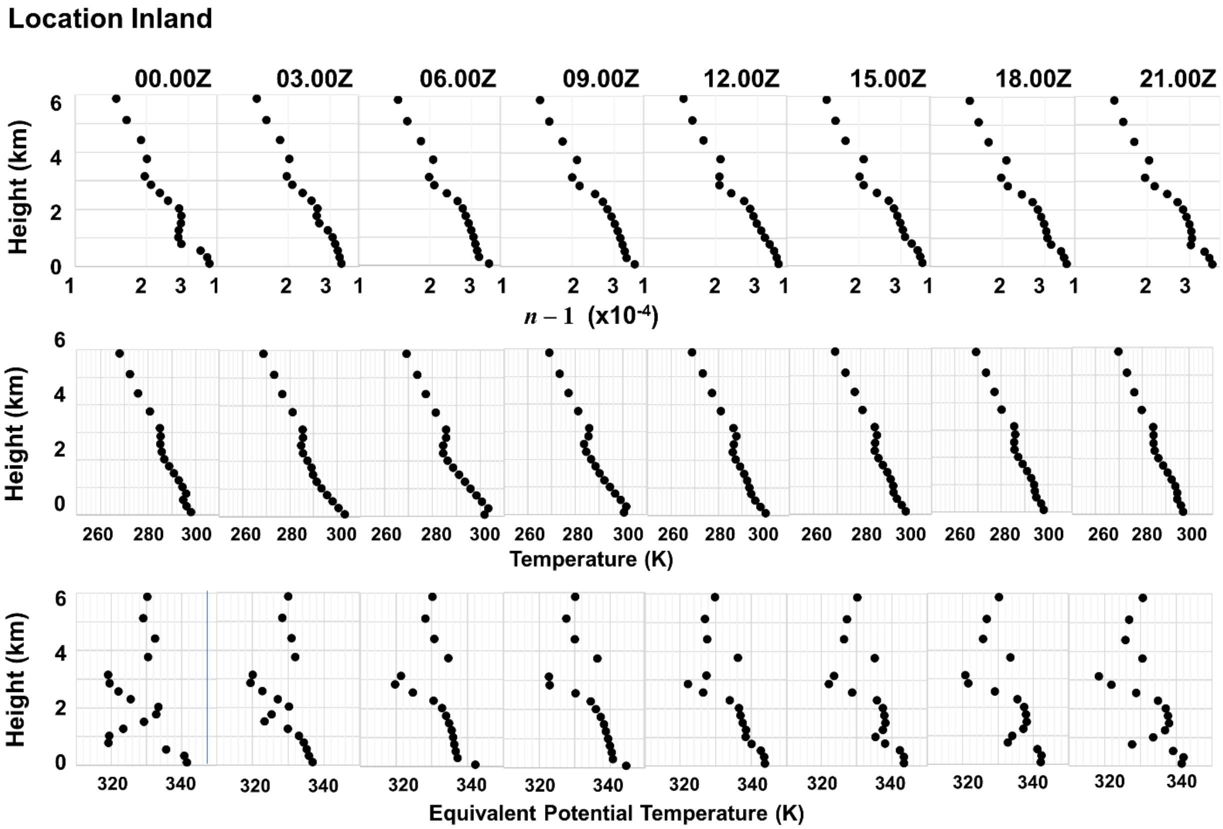

Figure S4. Diurnal change in height distributions of $(n-1)$ in logarithmic scale, temperature and equivalent potential temperature at the inland location of Fig. S3. From 00Z to 21Z on 15 October 2015. (EXCEL 2019, Power Point 2019)

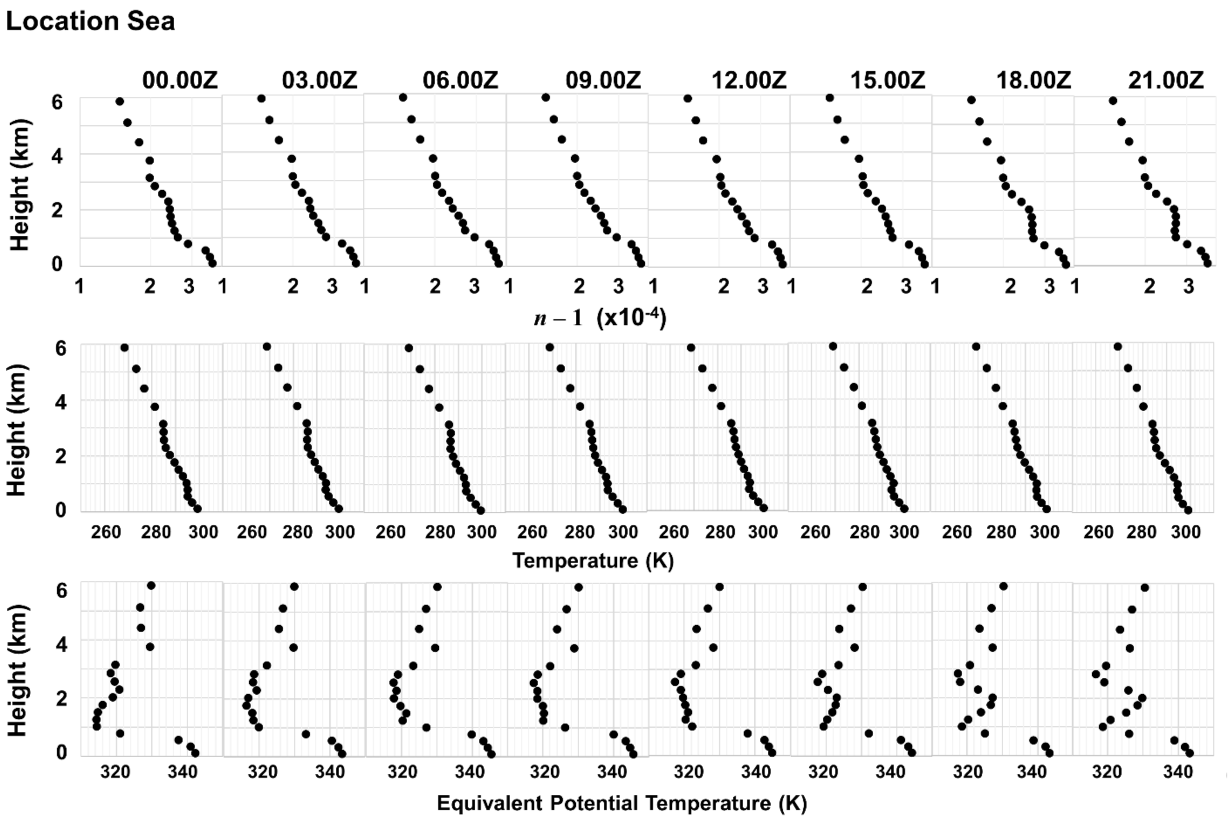

Figure S5. Diurnal change in height distributions of $(n-1)$ in logarithmic scale, temperature and equivalent potential temperature at the sea location of the map of Fig. S3. From 00Z to 21Z on 15 October 2015. (EXCEL 2019, Power Point 2019)

4. Air quality and visibility data in Palangka Raya. Air quality and visibility data of Palangka Raya (PKY) were obtained from AERONET (Level 2, http://aeronet.gsfc.nasa.gov) to determine the impact of fire smoke plumes. Figure S6 shows the temporal variation of the aerosol optical depth (AOD) obtained from the AERONET sun photometer installed at the Tjilik Riwut BMKG station of PKY. At two spectral wavelengths, 500 and 870 nm, the AODs that correspond to the aerosol concentration peaked at AOD = 6 during August–October 2015 due to peatland fires around PKY, while AODs in the wet season were below one. Figure S6 also shows the time series of AOD on 15 October 2015. For 01Z – 08Z, in which the AODs monotonically increased. After 08Z, the sun photometer stopped working due to the faint solar intensity diminished by the thick hazy air mass that influxes into PKY from the southern districts of Central Kalimantan.

Figure S7 shows that the visibility at the BMKG station ranged from 300 to 3,000 m. After the contaminated hazy air mass reached the PKY area from the southern district at 05Z, visibility decreased during 05Z – 08Z. This temporal variation corresponds to the increasing AOD for 05Z – 08Z as shown by the correlation diagram

of Fig. S6. A strong correlation (R^2 = 0.93) between one-hour averaged values of visibility and AOD for 00Z – 08Z indicates that we observed the hazy air flux at PKY.

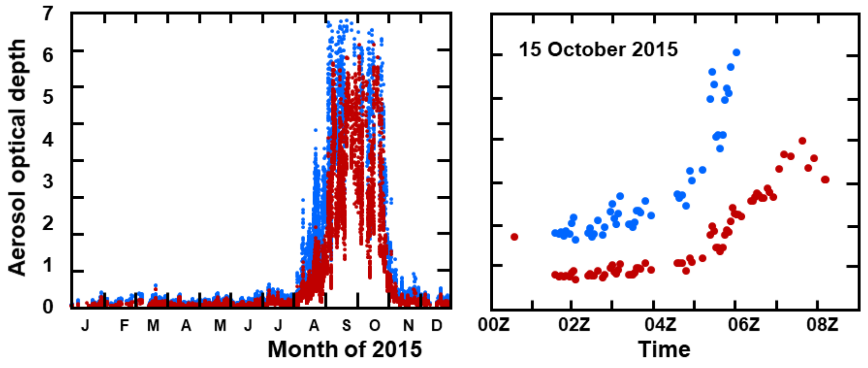

Figure S6. (Left) Timeseries of aerosol optical depth at 500 nm (blue) and 870 nm (red) from January to December 2015 at the Tjilik Riwut BMKG Station of Palangka Raya. (Right): Timeseries of AOD on 15 October 2015 from 01.30Z to 08.00Z. Data are provided by AERONET/NASA. (EXCEL 2019, Power Point 2019)

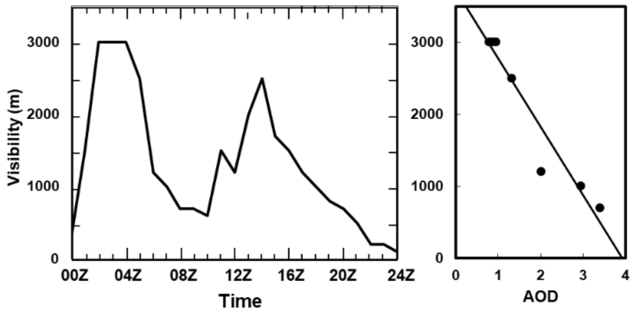

Figure S7. (Left) Time series of visibility at the Tjilik Riwut BMKG Station of Palangka Raya on 15 October 2015. Right: Correlation diagram between one-hour average values of visibility and aerosol optical depth (AOD) at 870 nm in Fig. S6 for 00Z – 08Z. R^2 = 0.93. (EXCEL 2019, Power Point 2019)

5. Diurnal cycle of temperature and humidity

Figure S8 shows three-hr time-average values of temperature and humidity at Palangka Raya Airport on Oct. 15th, 2015. At 21Z (04LT), humidity is > 90% and temperature < 25°C. As soon as the sun rises at 00Z (07LT), humidity goes down. Under the high humid condition at midnight, the smaller nuclei of aerosols grow to larger PMs by condensation of water onto them, which reduce visibility by increasing scattering ability. Weather data were available on http://aviation.bmkg.go.id/web/station.php?c=ID&pn=0.

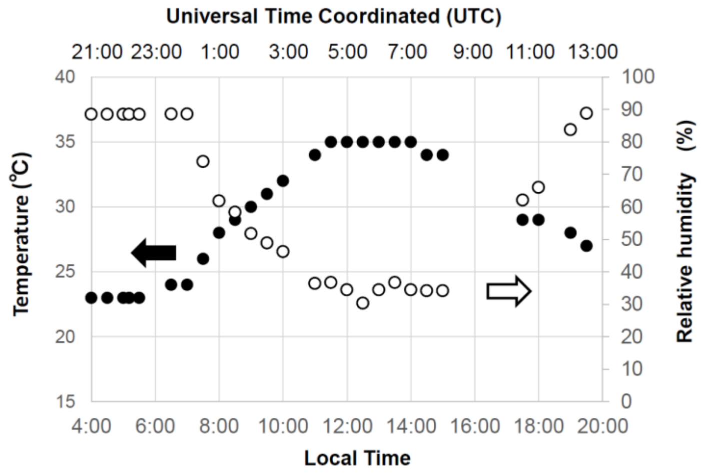

Figure S8. Time series of temperature and humidity at Palangka Raya Airport on Oct. 15th, 2015. (EXCEL 2019, Power Point 2019)
